# Angiopoietin-like 4 in glucocorticoid induced insulin resistance

**DOI:** 10.18632/oncotarget.21294

**Published:** 2017-09-28

**Authors:** Rebecca A. Lee, Jen-Chywan Wang

**Affiliations:** Jen-Chywan Wang: Endocrinology Graduate Program and Department of Nutritional Sciences & Toxicology, University of California, Berkeley, CA, USA

**Keywords:** glucocorticoid, angiopoietin-like 4, ceramide, ceramide synthase, insulin resistance

Glucocorticoids play a key role in metabolic adaptation during stress. The major metabolic function of glucocorticoids is to preserve plasma the glucose levels that serve as the major energy source for the brain [1]. Therefore, glucocorticoids antagonize the ability of insulin to suppress hepatic gluconeogenesis and promote glucose utilization in white adipose tissue (WAT) and skeletal muscle [1]. Thus, it is not surprising that chronic and/or excess glucocorticoid exposure causes insulin resistance. Glucocorticoids convey their signals mainly through an intracellular glucocorticoid receptor (GR), which functions as a transcription factor. Hence, the primary target genes of GR initiate the physiological and the pathophysiological responses of glucocorticoids. Identifying GR primary targets that are involved in glucocorticoid-induced insulin resistance will not only advance our understanding of the mechanisms governing glucocorticoid-induced insulin resistance, but also provide specific targets to improve insulin sensitivity.

**Figure 1:**
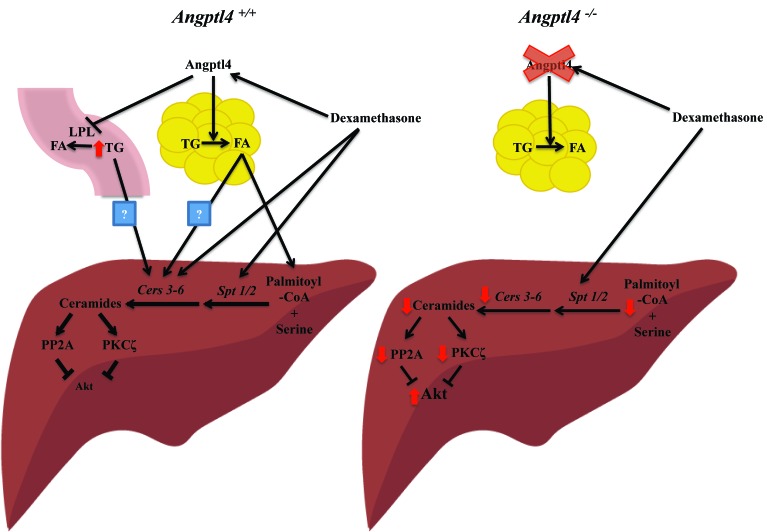
Model of glucocorticoid-induced hepatic insulin resistance In Angptl4 expressing mice (*Angptl4*+/+), dexamethasone (a synthetic glucocorticoid) induces the expression of genes encoding enzymes in ceramide synthesis, such as *Spt1/2* and *Cers3-6*, to increase hepatic ceramide production, which activates PP2A and PKCζ to inhibit Akt to cause hepatic insulin resistance. In *Angptl4* null mice (*Angptl4*-/-), the ability of dexamethasone to augment *Cers3-6* expression is impaired, which results in reduced ceramide levels and lower PP2A and PKCζ activity. As a result, hepatic insulin sensitivity is improved. Angptl4 stimulates lipolysis in white adipose tissue and also inhibits lipoprotein lipase (LPL) activity in circulation. It is unclear which of these functions is responsible for its action in glucocorticoid-induction hepatic ceramide production.

Recently, we have shown that glucocorticoid-induced insulin resistance requires participation of a GR primary target gene, *angiopoietin-like 4* (*Angptl4*) [2]. Angptl4 encodes a secreted protein that inhibits extracellular lipoprotein lipase (LPL) [3] but promotes lipolysis in adipocytes [4]. Angptl4, which is induced by glucocorticoids, promotes the production of ceramides in the liver, which then activates protein phosphatase 2A (PP2A) and protein kinase c ζ (PKCζ) to suppress hepatic insulin signaling [2] (Fig. 1). Glucocorticoid treatment elevates expression of genes encoding enzymes in the ceramide biosynthetic program, such as *serine palmitoyltransferase 1* and *2* (*Spt1/2*) and *ceramide synthase 3-6* (*Cers3-6*). In *Angptl4* null mice (*Angptl4*^*-/-*^), the ability of glucocorticoids to induce expression of *Cers3-6* is impaired whereas their effect on *Spt1/2* is maintained [2]. These results suggest that one of Angptl4’s roles in glucocorticoid-induced insulin resistance is to augment hepatic *Cers3-6* expression. What is the mechanism underlying Angptl4 action on inducing hepatic *Cers3-6* gene expression? It has previously been shown that Angptl4 is required for glucocorticoid-induced adipocyte lipolysis [4]. Therefore, we speculate that Angptl4 mediates the glucocorticoid effect on WAT lipolysis, thereby generating fatty acids that are then mobilized to liver to induce signals that directly or indirectly participate in promoting *Cers3-6* expression (Figure 1). Previous studies have shown that lipolysis in WAT is involved in glucocorticoid-induced insulin resistance [1]. Activation of lipolysis also provides palmitate, the precursor of *de novo* ceramide synthesis. Nonetheless, the potential role of LPL, which is inhibited by Angptl4, in glucocorticoid-induced insulin resistance has not been excluded and needs to be further studied (Figure 1).

Several questions are yet to be resolved. First, although the importance of ceramide production in glucocorticoid-induced insulin resistance is supported by this as well as by a previous report [5], it is unclear which ceramide specie(s) are required for this glucocorticoid response. Distinct ceramide synthases are responsible for the synthesis of different ceramide species. This report shows that glucocorticoids increase multiple ceramide species and induces at least 4 ceramide synthases, *Cers3-6* [2]. Intriguingly, various ceramide species and ceramide synthases have been shown to play opposing roles in regulating insulin sensitivity [6]. For example, deleting *Cers6* protects mice from diet induced obesity and glucose intolerance. In contrast, haploinsufficiency of *Cers2* exacerbates diet-induced insulin resistance. Thus, it is critical to determine which specific ceramide synthase(s), *Cers3-6*, mediate the inhibitory effects of GC on insulin action in the liver. In fact, it is unclear whether all or only certain ceramide synthases and ceramide species activate PP2A and/or PKCζ. Moreover, sphingosine 1 phosphate (S1P) levels and *sphingosine kinase 1* (*Sphk1*) expression in the liver are also elevated by glucocorticoids, which is attenuated in *Angptl4*^*-/-*^ mice. Whether S1P and Sphk1 are also involved in glucocorticoid-induced insulin resistance needs to be determined.

Second, in addition to the liver, insulin resistance in skeletal muscle is also reversed in *Angptl4*^*-/-*^ mice. However, the ceramide levels in gastrocnemius muscle are unchanged upon glucocorticoid treatment. In fact, targeted metabolomics studies in this report found only 9 metabolites to be modulated by glucocorticoid treatment with none of them being known to suppress insulin signaling. Notably, plasma ceramide levels are elevated by glucocorticoids, which is diminished in *Angptl4*^*-/-*^ mice. Is it possible that excess ceramides produced in the liver are mobilized to skeletal muscle to cause insulin resistance? Alternatively, Angptl4-dependent, but ceramide-independent, mechanisms may mediate glucocorticoid induced insulin resistance in skeletal muscle.

Finally, how does Angptl4 promote the expression of *Cers3-6* genes in the liver? Chromatin immunoprecipitation sequencing of mouse liver showed that both *Cers3* and *Cers6* contain GR binding regions in their genomic regions [7]. Thus, they are potential primary targets of GR, although this notion will need to be further examined. In any case, Angptl4 is required to potentiate glucocorticoid action in promoting expression of these two genes. In contrast to Cers3 and Cers6, GR binding regions are not found in nearby *Cers4* and *Cers5* genomic regions. For the latter case, their expression could be stimulated by the direct effects of Angptl4 on hepatocytes or by the indirect effects of Angptl4 on adipocytes or other cell types.

In summary, this report has established the key role of Angptl4 in ceramide-initiated signaling in mediating glucocorticoid-induced hepatic insulin resistance. As Angptl4 is a circulating factor, it would be possible to examine whether Angptl4 antibodies can reduce not only chronic or excess glucocorticoid-induced insulin resistance, but also lipid disorders, since Angptl4 has been shown to be required for glucocorticoid-induced hepatic steatosis and hypertriglyceridemia [8].
